# Recognition of Impact Load on Connecting-Shaft Rotor System Based on Motor Current Signal Analysis

**DOI:** 10.3390/s24217008

**Published:** 2024-10-31

**Authors:** Kun Zhang, Zhaojian Yang, Qingbao Bao, Jianwen Zhang

**Affiliations:** 1 College of Mechanical Engineering, Taiyuan University of Technology, Taiyuan 030024, China; 2 Postdoctoral Science Research Workstation, Taiyuan Heavy Machinery Group Co., Ltd., Taiyuan 030024, China; 3 College of Mathematics, Taiyuan University of Technology, Taiyuan 030024, China

**Keywords:** motor current, feature extraction, rotor system, load recognition

## Abstract

Impact loads affect the operational performance and safety life of rolling equipment’s connecting-shaft rotor system, even causing faults and accidents. Therefore, recognizing and investigating impact loads is of great significance. Hence, a load recognition method based on motor current information is proposed in this paper to recognize impact loads on the connecting-shaft rotor system. First, the fast Fourier transform is used to obtain the frequency domain information for the motor’s current response signal from the rotor system load recognition test. Consequently, the required load response information can be presented more clearly using the singular value decomposition method to remove the power frequency components in the current signal. Then, wavelet packet decomposition is performed on the signal to generate energy analysis feature vectors. A qualitative recognition of the impact load on the system is achieved by learning vector quantization neural networks; the resulting load recognition results are good. These findings indicate that using the motor current as the analysis signal can solve the problem of the difficult layout for traditional vibration sensors in rolling sites. The preprocessing and recognition method of the current response signal can recognize the impact load, confirming the applicability and feasibility of the proposed method.

## 1. Introduction

The rolling industry, as a key foundational sector, directly embodies a country’s economic strength and comprehensive national strength. The improvement in its production efficiency, product quality, and equipment reliability is a significant aspect in enhancing a nation’s industrial competitiveness. During the production process, the connecting-shaft rotor system might encounter sudden loads, which are completely different from stable loads experienced during constant speed operation. Impact loads, being common and challenging working conditions, not only cause additional vibrations and noise, but also can lead to a decline in equipment performance, accelerate fatigue damage, and shorten equipment’s lifespan [1]. Moreover, impact loads may also affect the structural integrity of equipment, such as causing vibration or damage to the system and its components, thereby increasing maintenance costs and downtime. More seriously, strong impact loads may affect product quality and even cause safety accidents, posing serious threats to production and personnel safety [2].

In this context, recognizing and analyzing impact loads on the connecting-shaft rotor system holds great significance. This process helps staff quickly identify potential issues and plays a critical role in implementing preventive maintenance. It can also predict and avoid unforeseen downtime events, ensuring stable industrial production. Engineering designers can optimize the rotor and its supporting structures through such analysis, enhancing the impact resistance, thereby prolonging the equipment’s service life. Particularly during the design of new rotor systems, impact response testing is an essential means to evaluate the effectiveness of the design, ensuring the equipment operates correctly under anticipated working conditions and ultimately improving the system’s reliability and safety.

There are generally two ways to determine loads in practical engineering: direct measurement and load recognition [3]. However, the first approach is often difficult to implement due to the constraints of the production environment. Therefore, determining loads is a challenge that needs to be addressed through load recognition methods [4,5]. “Load recognition” involves the second type of inverse problem in dynamics. Load recognition starts from the output response and its characteristics, covering the load-excitation state, solving the original load acting on the system in reverse [6,7]. The key focus of load recognition lies in studying the non-stationary characteristics and the inversion of systems and signals. This involves exploring linear and nonlinear system theory, numerical computation methods, and computer simulation techniques. This topic has also sparked research interest in this field. For example, Xu et al. reviewed the development history of dynamic load recognition research in mechanical vibration [8]. Movahedian et al. investigated the problem of determining lateral loads on Kirchhoff plates [9]. Li et al. proposed a bridge load recognition method based on an improved fractional Tikhonov (IF-Tik) regularization method [10]. 

In the research of load recognition, traditional dynamic load recognition techniques primarily rely on frequency–domain methods and time–domain methods. The literature [11] delves deeply into the development and overview of frequency–domain recognition methods, which have had a relatively early start. Based on the assumption of system linearity, this method involves only the inversion of characteristic matrices, offering convenient dynamic calibration. However, it necessitates specific signal lengths, typically limiting its application to steady loads rather than nonlinear load issues. Moreover, around the resonance frequency, the system’s frequency response function can exhibit pathological properties, leading to an increase in recognition errors. In comparison, time–domain methods started later but exhibit a wider range of applicability since the recognition effects are not constrained by the signal sampling methods [12]. Nevertheless, time–domain methods are sensitive to the system’s initial conditions and boundary conditions, and their accuracy and robustness still require improvement [13]. With the advancement of computer technology, load recognition technology has also been increasingly developed. New recognition methods such as the inverse pseudo excitation method (IPEM), genetic algorithm (GA), and artificial neural network (ANN) have gradually become research focuses. Among these, the IPEM is a structural dynamics analysis technique, but it has a relatively weak anti-interference ability and low recognition accuracy [14]. The GA attracts attention for its concise operation process yet suffers from premature convergence, suboptimal global solutions, and poor convergence rates [15]. From an artificial intelligence perspective, the neural network method exhibits significant potential and prospects in handling dynamic load recognition tasks. Its main characteristics include an adaptability to dynamic loads, high recognition accuracy, good model stability, low data demand, and excellent noise resistance and robustness. Consequently, the neural network is an ideal tool for addressing load recognition problems such as nonlinearity and uncertainty [16]. Introducing neural network methods into the load recognition of rolling mill connecting-shaft rotor systems not only meets the demand for high-precision recognition in modern engineering technology, but also expands the application boundaries of artificial intelligence methods in the engineering field. Among them, one good example is the learning vector quantization neural network (LVQNN), an excellent intelligent algorithm. Its advantage lies in its simple network structure, which can handle complex classification tasks just through interactions among its internal units. It is also relatively easy to converge various complex and scattered design conditions to the conclusion [17,18]. Moreover, the LVQNN does not require the normalization or orthogonalization of input vectors during pattern recognition, making it more convenient and feasible. Therefore, the LVQNN has unique advantages in pattern recognition.

People often use vibration information to test and analyze signals when monitoring working conditions [19,20,21]. For example, Waheed et al. used an accelerometer to measure and analyze the vibration in additive manufacturing [22]. Shang et al. improved the performance of vibration energy harvesting from weak excitations by a lever-type mechanism [23]. Sharma et al. explored the monitoring and diagnosing of common faults in gearboxes from the frequency domain of vibration signals [24]. Due to the transmission effect within the system, the characteristics of the applied load can be reflected through the vibration information output by the system [25,26]. However, compared to traditional vibration signal analysis, motor current signature analysis (MCSA) within the field of electrical engineering possesses its own unique advantages [27]: (1) Non-contact measurement: vibration analysis typically requires the installation of sensors on the equipment. In contrast, MCSA only needs to be connected to the motor’s power line, without physical contact with the motor itself. (2) Signal effectiveness: in harsh operating conditions, vibration signals are often subject to noise interference, leading to difficulties in signal analysis and poor results. MCSA can avoid this issue by using current signals as the research object and is applicable to various AC induction motors in industrial settings. (3) Early warning capability: vibration analysis often can only accurately identify faults when they become more pronounced. MCSA is better at detecting subtle changes, enabling the early warning of potential issues. (4) Usability: vibration analysis requires specialized sensors, which are complex to install and can be constrained by environmental conditions, even making installation impossible [28]. MCSA only needs to be connected to the motor’s power line for data collection and analysis. This effect can be attributed to the working state of the internal components of the electric motor being directly reflected in the changes in the current in its stator [29,30]. The MCSA method originated from evaluating and diagnosing the working status and possible faults of the internal components of electric motors. With its continuous application and development, this method has gradually expanded to the fault assessment and research of connecting components outside the motor, such as gear friction and mechanical damage [31,32]. Gu et al. conducted in-depth research in this field [33]. The MCSA method was also used to measure the operating data of centrifugal pumps in reference [34]. Simultaneously, since the object of signal monitoring and research is the current variation in the stator in the motor, the acquisition of signals is relatively simple and feasible [35]. Therefore, due to the principle of electromagnetic action, when loads of different parameters are applied to the connecting-shaft rotor system, the air gap magnetic field inside the motor changes and can ultimately be reflected through the stator current. In this way, the load recognition of the connecting-shaft rotor system can be combined with the MCSA method.

In summary, through this literature research, the current status and development trends of the field are grasped, laying a theoretical foundation for load recognition research on connecting-shaft rotor systems. This leads to exploring new perspectives and interdisciplinary connections. It is found that modern load recognition techniques are rarely combined with studies on rolling mill connecting-shaft rotor systems, and even fewer studies focus on recognizing the impact loads suffered. The potential applications of artificial intelligence are promising, yet few reports exist on the incorporation of neural networks into load recognition for rolling mill connecting-shaft rotor systems. While MCSA in the electrical engineering domain boasts unique advantages, there is currently little research on the load recognition of connecting-shaft rotor systems based on MCSA both domestically and internationally. In comparison, there is a relatively abundant literature on using current to diagnose the motor itself. Based on the above analysis, the innovative entry point for this study has been established. Specifically, the advantages of MCSA are combined with the load recognition of the connecting-shaft rotor system driven by the motors to conduct relevant research. A new interdisciplinary intersection is established, proposing a qualitative recognition method for impact loads in rotor systems based on motor current information. Specifically, after a fast Fourier transform (FFT), the current frequency–domain information is preprocessed through singular value decomposition (SVD). This processing can weaken the power frequency component and cause the appearance of the required signal feature information. Once wavelet packet analysis (WPA) is used for the energy feature extraction, the qualitative recognition of the impact load in the rotor system is achieved through the LVQNN.

## 2. Methods and Principles

**Figure 1:**
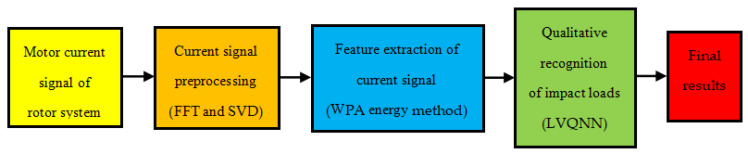
Qualitative load recognition process.

The entire process of the qualitative recognition method for the impact load of the connecting-shaft rotor system is shown in Figure 1. The column diagram shows the five steps of the method from left to right: importing motor current signals, the comprehensive preprocessing of the current signals, extracting the energy features of the current signals, the qualitative recognition of impact loads in the rotor system, and the output of the recognition results. The text within the parentheses in the diagram provides an explanation of the core steps, i.e., FFT and SVD, WPA energy feature extraction, and the qualitative recognition of impact loads in the rotor system using LVQNN.

### 2.1. Preprocessing Methods for Current Information

The air-gap magnetic field inside the motor changes accordingly due to the principle of electromagnetic action when different loads are applied to the rotor system. This phenomenon can ultimately be reflected through the current of the motor stator. Therefore, the current can characterize the load condition of the rotor system. Thus, the important information for load recognition is the current information of the motor stator. In this paper, the excellent MCSA method in the field of motor engineering is brought into the field of mechanical rotors, so the signal analysis object here is the motor current response signal. Given that traditional signal analysis primarily focuses on vibration signals, suitable preprocessing strategies for motor current characteristics must be sought. A short-time Fourier transform (STFT) can only handle signals with stationary changes due to its use of a constant window size for the signal analysis [36]. A FFT is an efficient signal processing algorithm with a wide range of applications. It can accommodate more complex sampling patterns, offering greater flexibility when handling signals in various practical applications [37]. Principal component analysis (PCA) computes efficiently by decomposing the eigenvalues of the covariance matrix. But, for nonlinear data, PCA often performs worse than SVD. A wavelet transform (WT) offers a good balance in time–frequency resolution but is constrained by its signal energy leakage and non-adaptability. Compared to wavelet decomposition, WPA can better reflect the full frequency characteristics of signals and provide a higher frequency resolution [38]. Based on these comparative analyses, this paper proposes a novel integrated signal preprocessing method for current signals, combining FFT, SVD, and WPA. This preprocessing method combines efficient the frequency–domain analysis of the FFT, flexible noise/dimension reduction in SVD, and multi-scale fine analysis of WPA. This scheme aims to integrate the advantages of each method while mitigating their individual limitations, thereby enhancing their overall signal processing performance and providing a more reliable foundation for subsequent load recognition.

#### 2.1.1. Fast Fourier Transform

The FFT is an efficient signal processing algorithm to compute the discrete Fourier transform (DFT) [37]. The FFT was first developed by J W. Cooley and T W. Tukey. The FFT achieves a butterfly iteration operation by utilizing rotation factors’ periodicity, symmetry, and scalability. Specifically, the FFT is based on decomposing DFT calculations into smaller DFT ones and using the combination of these smaller results to obtain the final result. The main advantage of this method is that the time complexity of calculating the DFT can be reduced from *O* (N^2^) to *O* (N × logN) for signals of length N, significantly reducing the computational load of the DFT [39].

The basic principles of the FFT are briefly explained, starting with the DFT operations.

The basic operation of the DFT is as follows:

(1) $$X(k)=DFT[x(n)]=\sum_{n=0}^{N-1}x(n)W_{N}^{kn},\;\;\;\;\;\;\;\;\;\;k=0,1,\ldots ,N-1$$

(2) $$x(n)=IDFT[X(k)]=\sum_{n=0}^{N-1}X(k)W_{N}^{-kn},\;\;\;\;\;\;\;\;\;k=0,1,\ldots ,N-1$$

When calculating the DFT using this method, 4N real multiplications and (4N − 2) additions are required for each *k* value of *X*(*k*). Hence, a total of 4N × N multiplications of real numbers and (4N − 2) × N additions of real numbers are required for the N values of *k*.

The basic equation of the FFT is as follows:

(3) $$A_{k}=\sum_{n=0}^{N-1}a_{n}W_{N}^{kn},\;\;\;\;\;\;\;\;\;\;k=0,1,\ldots ,N-1$$

where *a_n_* is the sample value of the time–domain signal and $W_{N}^{kn}$ is a complex exponential function used to calculate values in the frequency domain. 

Equation (3) describes the core calculation process of the FFT algorithm and improves the DFT algorithm, reducing the computational load. The basic idea of the FFT is utilizing the periodicity and symmetry of $W_{N}^{kn}$ in the DFT to transform the entire DFT calculation into a series of iterative operations. Hence, the computational process is significantly improved [40].

#### 2.1.2. Singular Value Decomposition

Various interferences in its operation often cause unnecessary components to be mixed into the rotor system’s response signals, affecting the effective signal’s quality [41,42]. Therefore, it is necessary to process these signals. The basic idea of the SVD method is retaining the singular value parts that exceed a specific threshold and reconstructing the original signal. Therefore, the SVD method can remove noise and improve the signal purity [43]. The relevant literature has also confirmed the effectiveness of using the SVD method for signal processing [44,45].

Singular value decomposition is an important matrix decomposition method that can decompose a complex matrix into the product of three simple matrices, simplifying matrix operations [46,47]. Specifically, for any m×n-order matrix A, there exists a decomposition:

*A* = *UWV*^T^ (4)

Establishing the attractor matrix is crucial in the SVD process and can be obtained by utilizing the original signal transformation. A digital signal consisting of N sampling points is treated as a time series $X=\left[x_{1},x_{2},\cdots x_{N}\right]$, and an m×n attractor matrix is constructed based on this vector. Let $y_{1}=\left[x_{1},x_{2},\cdots ,x_{n}\right]$ be the first row of the attractor matrix with *n* columns. The second row $y_{2}=\left[x_{2},x_{3},\cdots ,x_{n+1}\right]$ is constructed by shifting *y*_1_ one step to the right. This process is repeated until the last row $y_{m}=\left[x_{m},x_{m+1},\cdots ,x_{n+m-1}\right]$ is constructed. Therefore, it can be obtained as follows:

(5) $$A={\left[\begin{array}{cccc} y_{1} & y_{2} & \cdots & y_{m} \end{array}\right]}^{T}=\left[\begin{array}{cccc} x_{1} & x_{2} & \cdots & x_{n}\\ x_{2} & x_{3} & \cdots & x_{n-1}\\ \cdots & \cdots & \cdots & \cdots \\ x_{m} & x_{m+1} & \cdots & x_{n+m-1} \end{array}\right]$$

Another approach is to continuously perform an equal length truncation on the signal. A digital signal consisting of N sampling points is treated as a time series $X=\left[x_{1},x_{2},\cdots x_{N}\right]$. Let $z_{1}=\left[x_{1},x_{2},\cdots ,x_{n}\right]$ be the first row of the attractor matrix *A*. The second row $z_{2}=\left[x_{n+1},x_{n+2},\cdots ,x_{2n}\right]$ is constructed by equidistant cutting. This process is repeated until the last row $z_{m}=\left[x_{(m-1)n+1},x_{(m-1)n+2},\cdots ,x_{mn}\right]$ is constructed. Therefore, it can be obtained as follows:

(6) $$A=\left[\begin{array}{cccc} z_{1} & z_{2} & \cdots & z_{m} \end{array}\right]=\left[\begin{array}{cccc} x_{1} & x_{2} & \cdots & x_{n}\\ x_{n+1} & x_{n+2} & \cdots & x_{2n}\\ \cdots & \cdots & \cdots & \cdots \\ x_{\left(m-1\right)n+1} & x_{\left(m-1\right)n+2} & \cdots & x_{mn} \end{array}\right]$$

This decomposition process reveals the intrinsic structure of the matrix *A*, where *A* is a *m* × *n* original matrix, *U* is an *m* × *m* orthogonal matrix, and its column vector is the left singular vector of the original matrix *A*. These vectors make up the orthogonal basis of the input space. The matrix *W* is an *m* × *n* semi-positive definite diagonal matrix, and the elements on the diagonal are the singular values of *A*. These values are non-negative and usually arranged in descending order. The matrix *V* is an *n* × *n* orthogonal matrix, and its column vectors are the eigenvectors of *A*^T^*A*. The matrix *V*^T^ is the conjugate transpose of the *n* × *n* orthogonal matrix *V*, whose column vector is the right singular vector of the original matrix *A*. These vectors make up the orthogonal basis of the output space.

In summary, SVD provides an effective data analysis method by extracting the main data features. SVD is useful for data dimensionality reduction, signal processing, and recommendation systems [48].

#### 2.1.3. Wavelet Packet Energy Feature Extraction

The wavelet packet energy method (WPEM) is based on wavelet packet decomposition (WPD), a time–frequency localization analysis method [49]. WPD is a recursive decomposition of signals into high and low frequencies. This decomposition method is an improvement on wavelet decomposition [50]. Simultaneously, WPD is characterized by time–frequency localization, enabling it to adaptively select frequency bands. This processing method is more refined and comprehensive compared to traditional wavelet decomposition. Consequently, WPD can better reflect the full frequency characteristics of the signal [38]. In addition, WPD somewhat avoids the noise interference that may be introduced in traditional spectrum analysis. Therefore, WPD can extract the information contained in the signal in a more effective way [51,52,53].

**Figure 2:**
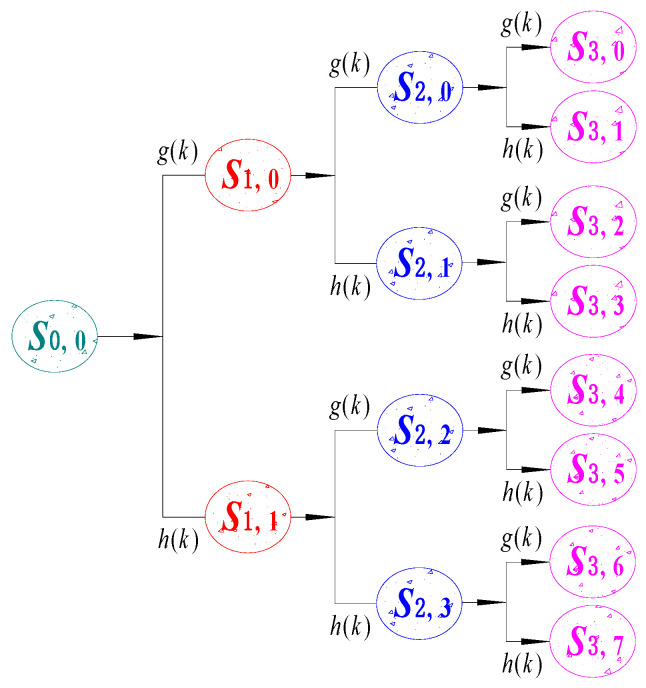
The structure diagram of WPD (three-layer). The leftmost original signal *S*_0,0_ is decomposed layer by layer to the rightmost side.

Figure 2 shows the process of three-layer WPD. 

The elliptical shape represents *S_i,j_*, or the decomposed signal corresponding to the *j*th node of the *i*-th layer (i.e., the degree of measurement). For example, *S*_0,0_ are the original signals before decomposition, which are then decomposed layer by layer in the direction of the arrows in the figure. Equation (7) can be obtained as follows:

(7) $$\begin{array}{l} S_{0,0}=S_{1,0}+S_{1,1}=S_{2,0}+S_{2,1}+S_{2,2}+S_{2,3}\\ =S_{3,0}+S_{3,1}+S_{3,2}+S_{3,3}+S_{3,4}+S_{3,5}+S_{3,6}+S_{3,7} \end{array}$$

If the number of nodes *j* is even, the signal corresponding to that node is the low-frequency component obtained through the low-pass filter *g*(*k*); if *j* is odd, the signal corresponding to that node is the high-frequency component obtained through the high-pass filter *h*(*k*). The high-pass and low-pass filter coefficients need to satisfy the orthogonal relationship of Equation (8):

(8) $$g\left(k\right)={\left(-1\right)}^{k}h\left(1-k\right)$$

The decomposed signals obtained from different decomposition layers can be calculated layer by layer according to Equations (9) and (10):

(9) $$S_{i+1,2j}\left(n\right)=\sum_{k}g\left(k-2n\right)S_{i,j}\left(k\right)$$

(10) $$S_{i+1,2j+1}\left(n\right)=\sum_{k}g\left(k-2n\right)S_{i,j}\left(k\right)$$

According to the above decomposition method, the signal will obtain 2*^i^* feature signals after the *i*-th layer WPD, each matching the corresponding frequency band.

Each WPD divides the signal frequency band and obtains the signal components of the corresponding sub-bands. Hence, the characteristic information of the original signal is also present in each sub-band signal [54]. The characteristics of each signal in the frequency domain can be analyzed and recognized by the energy of each frequency band. Therefore, after the WPD of the signal, the energy distribution characteristics in different frequency bands can be used as an important basis for signal recognition.

According to Parseval’s theorem, the total signal’s energy in its time domain equals the total energy in its frequency domain. Only the high-frequency and low-frequency components of the signal are separated for WPD. The signal form changes, but the total energy before and after decomposition remains equal. If a signal *x*(*t*) is subjected to a three-layer wavelet packet solution, the energy value $E_{3j}$ at node $\left(3,j\right)$ is expressed as Equation (11), and *k* represents the sampling point:

(11) $$E_{3j}=\sum_{k=1}^{n}x_{jk}^{2}$$

The energy percentage of this node is

(12) $$P_{j}=E_{3j}/\sum_{j=0}^{7}E_{3j}$$

The eigenvector *P* can be expressed by taking the energy percentage of each sub-band as the eigenvector of the current signal:

(13) $$P=[P_{0},P_{1},P_{2},P_{3},P_{4},P_{5},P_{6},P_{7}]$$

Based on the energy distribution of the motor current under different load excitations, the correlation between the energy percentage changes and load parameters can be indirectly revealed. Specifically, WPD is applied to the current response signals under different load conditions, and the energy distribution of each decomposition node is compared and analyzed to understand the dynamic changes in the system energy under load conditions. By utilizing the energy distribution characteristics within each frequency band, support and basis can be provided for recognizing and judging the specific attributes of the excitation load.

### 2.2. LVQNN Screening Method

#### 2.2.1. Network Structure

The LVQNN is a supervised learning statistical classification algorithm from prototype clustering [55]. The LVQNN evolved from the Kohonen competitive algorithm and can be used as an input feedforward neural network with supervised learning methods for training competitive layers [56]. 

**Figure 3:**
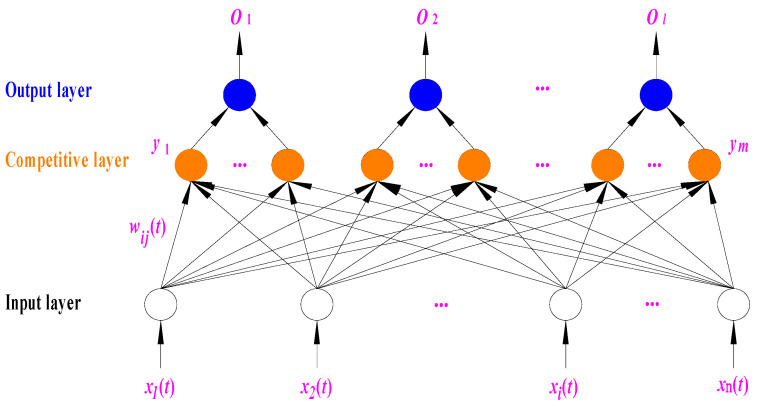
The network topology diagram for the LVQ process. Neurons are divided into the input layer, competition layer, and linear output layer from bottom to top.

The LVQNN comprises three parts of neurons: the input, competition, and linear output layers [57,58]. Figure 3 depicts a schematic diagram of the neural network topology for learning the vector quantization process, where the small circles represent neurons. Neurons of the same color belong to the same level. For example, the orange circles represent neurons in the competitive layer. The input layer and the competition layer are fully connected. In contrast, the competition and linear output layers are partially connected.

#### 2.2.2. Learning Algorithm

The LVQNN algorithm is an effective learning method for competitive layer training in the presence of teachers, improving the self-organizing feature mapping algorithm [59,60]. The LVQNN can be divided into the LVQ1 and LVQ2 algorithms. The update rules of the LVQ2 are more complex than the LVQ1. Furthermore, the LVQ2 can better handle situations where the boundaries are blurred. Hence, the LVQ2 often produces better computational results.

The algorithms are briefly described as follows:

- LVQ1 algorithm.

Step 1: Initialize the weight $w_{ij}$ and learning rate *η* (*η* > 0) between the input layer and the competition layer. 

Step 2: Assign the input vector $x={\left(x_{1},x_{2},\cdots ,x_{R}\right)}^{T}$ to the input layer. The distance value *d* between the input vector and the competing layer neurons can be obtained using the following equation. Among them, the weights of the two are represented by $w_{ij}$.

(14) $$d_{i}=\sqrt{\sum_{j=1}^{R}{\left(x_{i}-w_{ij}\right)}^{2}}\;\;\;\;\;\;\;\;\;\;\;\;\;\;\;\;\;i=1,2,\cdots ,S^{1}$$

Step 3: For the linear input layers, if *d* = *d_i_* is minimized, then the label *C_i_* represents the neuron category.

Step 4: For the input vector, *C_x_* is used to indicate its category.

When $C_{i}=C_{x}$, the weight correction equation is as follows:

(15) $$w_{ij\_new}=w_{ij\_old}+\eta \left(x-w_{ij\_old}\right)$$

On the contrary, the weight equation adopts the following formula:

(16) $$w_{ij\_new}=w_{ij\_old}-\eta \left(x-w_{ij\_old}\right)$$

- LVQ2 algorithm.

Step 1: The LVQ1 algorithm is used to learn all the input patterns.

Step 2: The input vector $x={\left(x_{1},x_{2},\cdots ,x_{R}\right)}^{T}$ is assigned to the input layer, and Equation (14) is used to obtain d, i.e., the distance between the input vector and the competing layer neurons. 

Step 3: When the distance *d* between adjacent competing neurons is minimized, they are denoted as *i* and *j*.

Step 4: If *C_i_ = C_x_*, the weights between neurons *i* and *j* need to be adjusted in the following way:

(17) $$\left.\begin{array}{l} {w_{i}}^{new}={w_{i}}^{old}+a\left(x-{w_{i}}^{old}\right)\;\\ {w_{j}}^{new}={w_{j}}^{old}-a\left(x-{w_{j}}^{old}\right) \end{array}\right\}$$

If *C_j_ = C_x_*, the weights of neurons *i* and *j* need to be adjusted in the following way:

(18) $$\left.\begin{array}{l} {w_{i}}^{new}={w_{i}}^{old}-a\left(x-{w_{i}}^{old}\right)\;\\ {w_{j}}^{new}={w_{j}}^{old}+a\left(x-{w_{j}}^{old}\right) \end{array}\right\}$$

Step 5: If neurons *i* and *j* fail to meet the conditions of step *d*, only the neuron closest to the input vector needs to be updated.

#### 2.2.3. Model Establishment

**Figure 4:**
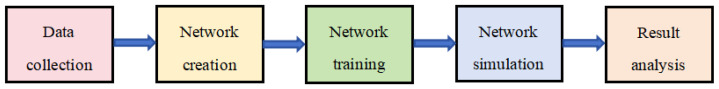
Design step flow chart.

The LVQNN model’s design steps are shown in Figure 4. From left to right, it is mainly divided into five steps: data collection, network creation, network training, network simulation, and result analysis.

The data processed through signal preprocessing can be utilized for the model analysis. After the data are ready, the LVQNN is constructed by calling the function *newlvq*() in MATLAB. After the network is created, the training set data can be input into the network to train it. After the network is trained, the corresponding output results can be obtained by inputting the test data into the network. The effectiveness of this method can be evaluated through the analysis of network simulation results.

## 3. Results and Analysis

### 3.1. Measurement and Acquisition of Motor Current Signal

This paper is based on the motor’s current response information for recognizing loads on the connecting-shaft rotor system. The response signals come from the load recognition test. The rotor system test bench involved in this experiment can simulate the operating conditions of the rotor system through dynamic torque loading with different parameters under different working conditions. Hence, dynamic torque loading modes under different parameters were designed and implemented to reproduce the loading conditions of the rotor system during normal operation (stead load) and external impact.

**Figure 5:**
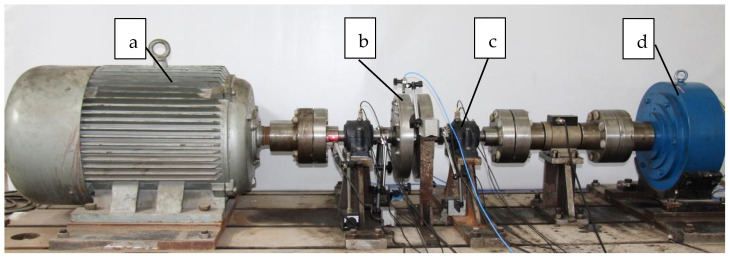
The main body of the load recognition test bench. (**a**) Three phase asynchronous motor (connected to current transformers to measure motor current); (**b**) rotor; (**c**) bearings; (**d**) magnetic powder brake.

The main experimental apparatus is shown in Figure 5. The main body of the test bench mainly includes a three-phase asynchronous motor (connected to a current transformer to measure the motor current), rotor, bearings, and a magnetic powder brake. A three-phase asynchronous motor with a driving power of 55 kW and a rated speed of 1480 r/min is used.

**Figure 6:**
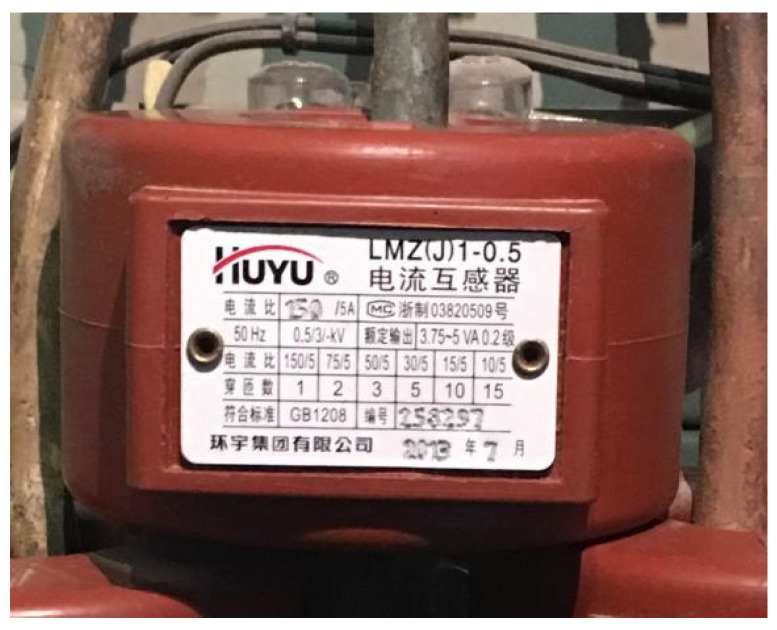
Current transformer. It is a device used to step down the current in a high-current circuit to a lower, more manageable level for measurement purposes.

Here, the motor current response signals are obtained by adding a current transformer (an electrical component used for measuring current) and connecting lines in the frequency conversion cabinet of the test bench. The employed current transformer is shown in Figure 6. After the motor current passes through the transformer, it can be connected to a testing instrument for signal pickup. The INV3060 tester is used with a sampling frequency of 2048 Hz. The original loaded impact loads can be recognized via this method in reverse based on the actual measured motor current response information.

### 3.2. Preprocessing the Motor Current Signal

#### 3.2.1. FFT Processing

**Figure 7:**
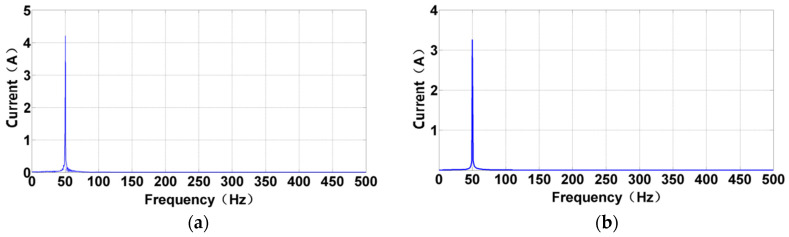
Spectrum of the stator current under impact load. The blue spectral line at the horizontal axis of 50 Hz indicates the significant influence of the power electricity. (**a**) Under 45 Nm impact load. (**b**) Under 30 Nm impact load.

External loads with different parameters acting on the connecting-shaft rotor system are investigated in this paper. Firstly, a fast Fourier transform is performed on the current response signals of the test motor corresponding to each load. Figure 7 shows the result after the FFT processing by taking the 45 Nm and 30 Nm impact load as an example. The signal converted to the frequency domain mainly exhibits the main frequency of 50 Hz, reflecting the frequency of power electricity. Moreover, other small frequency components can be seen around the main frequency. This observation indicates that the current response information caused by the impact load has been covered by the response information caused by the power electricity. Therefore, at this point, it is difficult to reflect the current response information corresponding to the load excitation solely from the FFT spectrum; hence, further signal extraction is required.

#### 3.2.2. SVD Processing

**Figure 8:**
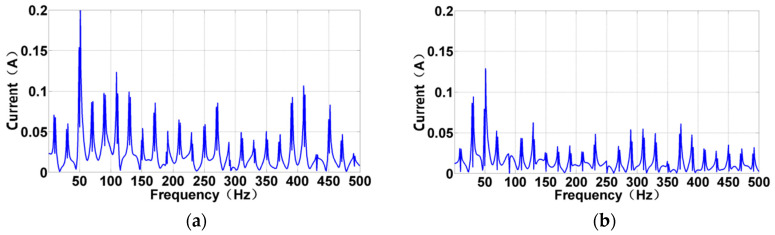
The current spectrum following SVD. It presents the spectral form after weakening the power electricity. (**a**) Under 45 Nm impact load. (**b**) Under 30 Nm impact load.

Figure 8 shows the SVD results of the corresponding current information under the impact load. The variation patterns of each frequency component are rich, significantly weakening the display effect of the 50 Hz main frequency in the previous step. Consequently, the load response information required for the research can be presented more clearly. Meanwhile, although SVD significantly weakens the frequency effect of dynamic electricity, a frequency effect of dynamic electricity can be observed in the spectrum.

#### 3.2.3. Energy Feature Extraction

The energy characteristics of the motor current information are extracted based on the calculation method in Section 2.1.3; the results are listed in Table 1, where the eight different frequency regions correspond to the bottom eight nodes.

**Figure 9:**
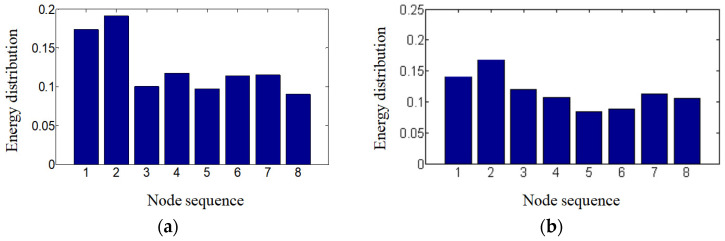
Node energy distribution under impact load. The energy distribution corresponding to the eight nodes is presented here. (**a**) Under 45 Nm impact load. (**b**) Under 30 Nm impact load.

The corresponding current response information of the rotor system under the impact load is obtained. The signal energy graph after preprocessing is obtained from this information and shown in Figure 9. The WPEM used here has significant advantages over the wavelet energy method (WEM) in its high-precision frequency analysis and energy distribution accuracy. The WPEM excels in complete multi-resolution analysis, which decomposes signals into various frequency sub-bands to more comprehensively analyze the signals’ spectral structure. By comparison, the WEM mainly focuses on the low-frequency part, and the high-frequency analysis is relatively rough, which cannot fully cover all frequency sub-bands. On the other hand, the WPEM can accurately distribute signal energy onto finer frequency sub-bands, providing a more detailed energy distribution map, which helps to accurately identify the energy gathering points and spectral characteristics. However, the WEM is relatively rough in its energy distribution, especially in the high-frequency part. This may potentially miss certain energy features.

### 3.3. Load Recognition

**Figure 10:**
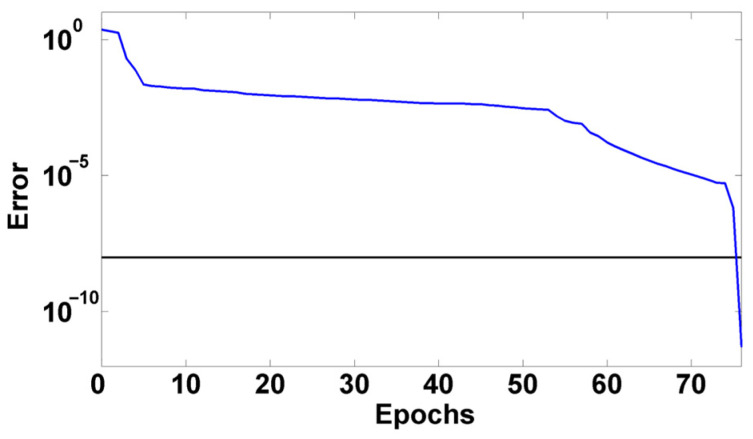
Network performance curve.

The LVQNN can be created by calling the *newlvq*() function in MATLAB. Since it is to explore the impact load relative to the steady state operation, the impact and steady load data of 20 × 2 × 8 different parameters in the shaft-connected rotor system test are randomly selected for the training, and the impact and steady load data of another 15 × 2 × 8 different parameters are used for the testing network. When establishing the LVQNN, if the dimension of the current energy characteristics mentioned above is eight, then the number of input neurons is eight. Since the load types to be recognized are “impact” and “steady”, the corresponding state numbers are “1” and “2”. The network is trained using MATLAB with a learning function of *learnlv*2 and a learning parameter LP of 0.01. The network performance curve at this point is shown in Figure 10. The model error rapidly decreases as the training progresses, indicating that the network model converges satisfactorily.

An example of training data is shown in Table 2, and an example of testing data is shown in Table 3. The training data are imported into the network. The network undergoes training and learning by using the function *train*(). The training sample does not overlap with the testing sample.

**Figure 11:**
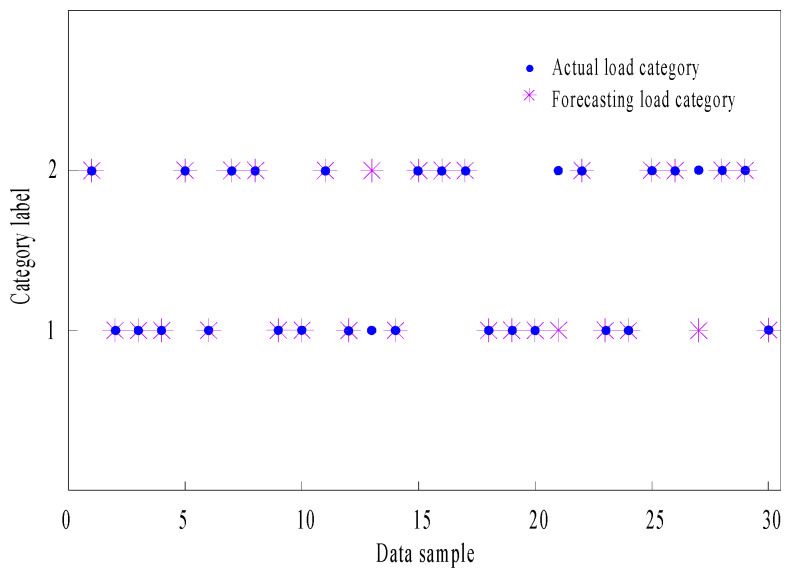
LVQNN prediction effect. The blue circle shape in the figure represents the actual load value, while the pink snowflake shape represents the predicted load value.

The function *sim*() of the LVQNN can be used to recognize the test set after pre-training. Figure 11 shows the prediction results of the LVQNN model. The true value of the load is represented by a “blue circle shape”. In contrast, the corresponding predicted value of the load is represented by a “pink snowflake shape”. The horizontal axis indicates different test data points. In the vertical axis, “1” represents the impact load state number, while “2” represents the steady load state number. According to the “pink snowflake shapes” and “blue circle shapes” in the figure, the data consistency between the two is good, indicating a high degree of matching between the predicted load results and the actual load values.

Figure 11 compares the load recognition results with the actual load values in the experiment. Subsequently, an error analysis is conducted, as shown in Table 4.

Table 4 shows that the recognition accuracy of the impact loads is relatively high, reaching 93.33%. This indicates that the method proposed in this paper is effective for recognizing impact loads. In comparison, the recognition accuracy for the steady loads is relatively lower at 86.67%, indicating that the algorithm performs better in handling impact loads than steady loads. The overall recognition accuracy is 90%, which means that the system has a relatively balanced performance in handling all loads. The result indicates that the network successfully recognized the input vector by testing the sample data. Therefore, the LVQNN can accurately recognize impact loads, indicating that the design of this network is reasonable. Due to time constraints, the recognition method proposed in this paper has not yet been integrated with the signal monitoring interface to achieve a fully closed-loop process from theory to practice. Therefore, further research is needed on online monitoring platforms to enable the real-time monitoring and recognition of impact loads on the connecting-shaft rotor system.

## 4. Conclusions

To effectively address the challenges of traditional sensor placement on rolling sites, this study explored how to apply the advantages of MCSA to load recognition in the shaft-rotor system driven by the motor, forming novel interdisciplinary research. By directly exploring the motor current response information, the impact load of the rotor system was successfully recognized. The main research content and conclusions of this paper are as follows:

Firstly, a preprocessing method for the current information of the connecting-shaft rotor system motor in response to the impact load excitation was proposed. After the FFT, the measured current frequency–domain signals used the SVD method to remove the power frequency component, making the characteristic frequency of the signal explicit. Then, the signal energy features were extracted through WPA and prepared for the subsequent payload recognition process.

Subsequently, a qualitative recognition method for rotor system impact load based on the motor current information was proposed. The test sample can qualitatively recognize the impact load of the rotor system through the trained LVQNN. Simultaneously, the load recognition results are good and meet practical requirements, confirming the applicability and feasibility of the proposed method. Understanding the behavior state of the connecting-shaft rotor system is deepened by recognizing and investigating the impact loads. Moreover, the equipment’s safety, reliability, and economy are improved. Lastly, the research provides theoretical and experimental support for system design optimization, operation monitoring, load analysis, and fault diagnosis. In the future, research will focus on online monitoring platforms for load recognition, as well as extending load recognition to more complex working environments.

## Figures and Tables

**Table 1:** Energy nodes.

Energy Node	Frequency Band (Hz)	Energy Node	Frequency Band (Hz)
(3, 0)	0~15.625	(3, 4)	62.50~78.125
(3, 1)	15.625~31.25	(3, 5)	78.125~93.75
(3, 2)	31.25~46.875	(3, 6)	93.75~109.375
(3, 3)	46.875~62.50	(3, 7)	109.375~125.0

**Table 2:** Example of training data.

Load	*P*_0_	*P*_1_	*P*_2_	*P*_3_	*P*_4_	*P*_5_	*P*_6_	*P*_7_
Impact	0.1568	0.1558	0.1381	0.0400	0.1505	0.1078	0.0734	0.1777
	0.0625	0.0363	0.3669	0.1959	0.0186	0.0124	0.2003	0.1072
	0.1597	0.1433	0.1204	0.1348	0.1119	0.1036	0.1191	0.1072
Steady	0.1040	0.1510	0.1065	0.1388	0.1624	0.0858	0.1457	0.1058
	0.1140	0.1565	0.1600	0.1024	0.1744	0.0439	0.1445	0.1044
	0.1255	0.0979	0.1012	0.1712	0.0778	0.1671	0.0771	0.1823

**Table 3:** Example of testing data.

Load	*P*_0_	*P*_1_	*P*_2_	*P*_3_	*P*_4_	*P*_5_	*P*_6_	*P*_7_
Impact	0.0636	0.0369	0.3662	0.1954	0.0187	0.0126	0.1997	0.1069
	0.1215	0.1149	0.1456	0.1530	0.1448	0.1791	0.0793	0.0620
Steady	0.0546	0.0266	0.3724	0.2009	0.0204	0.0119	0.2048	0.1084
	0.1074	0.1309	0.1309	0.2051	0.1697	0.0950	0.0703	0.0908

**Table 4:** Error analysis.

Error Analysis	Impact Loads	Steady Loads	All loads
Number of correct recognitions	14	13	27
Number of incorrect recognitions	1	2	3
Accuracy	93.33%	86.67%	90%

## Data Availability

The data were collected in the project being executed. Since the data are part of an ongoing study, the datasets presented in this article may not be readily available. Other instrument data used in this study are available in public domain resources.
